# Hierarchical Riblet Structures for Enhanced Drag Reduction and Broader Operational Range in Water Pipelines

**DOI:** 10.1021/acsestwater.5c00703

**Published:** 2025-09-05

**Authors:** Mirvahid Mohammadpour Chehrghani, Jamal Seyyed Monfared Zanjani, Doekle Yntema, David Matthews, Matthijn de Rooij

**Affiliations:** † Faculty of Engineering Technology, 3230 University of Twente, 7500AE Enschede, The Netherlands; ‡ Wetsus, European Centre of Excellence for Sustainable Water Technology, Oostergoweg 9, 8911 MA Leeuwarden, The Netherlands

**Keywords:** drinking water distribution, riblets, turbulent flow, pipe flow, biomimetic surfaces, frictional losses, energy efficiency

## Abstract

In drinking water distribution systems (DWDS), skin-friction drag in turbulent pipe flows contributes significantly to energy losses. Passive drag-reducing surfaces, such as shark-skin-inspired riblets, have shown promise in controlled environments but often underperform under variable flow conditions. This study addresses this limitation by developing and experimentally evaluating a Hierarchical Step-shaped Riblet (HSR) design aimed at sustaining drag reduction under the variable and fluctuating flow conditions typical of water pipelines. Building on conventional riblets (CR) and hierarchical riblets (HR), the HSR configuration introduces progressively tapered riblet tips to the hierarchical design to reduce shear-exposed surface area while maintaining effective interaction with vortices of varying size. Riblet designs were fabricated using high-resolution 3D printing and tested in variable flow conditions, simulating the flows in typical drinking water distribution networks. Drag reduction performance was evaluated across a Reynolds number range of 4200 to 20,000 using friction factor analysis and nondimensional riblet spacing. The HSR design achieved the highest peak drag reduction of 11.2% and sustained favorable performance across a broader range of flow conditions than the CR and HR designs. The results demonstrate that multiscale geometric tuning, combined with reduced shear exposure, enhances drag reduction across a broadened operational range suitable for drinking water distribution pipes.

## Graphical abstract

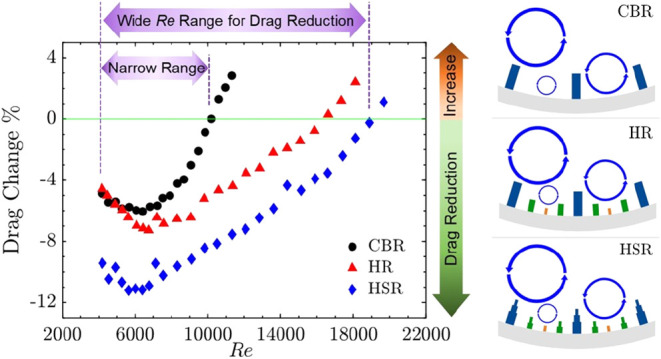

## 1 Introduction

Skin-friction drag in wall-bounded turbulent flows, such as those found in aircraft, maritime vessels, and pipelines, accounts for a substantial portion of total energy loss and operating costs.^1^ Therefore, even marginal reductions in drag can yield significant economic and environmental benefits by decreasing energy consumption.

Over recent decades, efforts to reduce turbulent drag have primarily focused on manipulating near-wall turbulence through active and passive control techniques.^2–5^ Active methods, such as oscillating walls,^6–8^ and gas injection,^9^ require external energy input, limiting their practicality in large-scale systems. In contrast, passive strategies involve surface modifications that reduce turbulence-induced friction without additional energy input. These include superhydrophobic coatings,^10–13^ dimpled and wavy surfaces,^14,15^ and bioinspired textures.^16–19^ Among passive strategies, shark-skin-inspired riblet surfaces have received the most attention and have shown the greatest promise for practical drag reduction.^20–25^ These surfaces feature flow-aligned, blade-like microstructures that interact with turbulent boundary layers to suppress near-wall vortices.^26^

While these designs have shown promising results in experimental studies, their performance in large-scale, real-world applications has been modest and the achieved performance remains significantly lower than the reductions of up to 10% typically reported in laboratory settings.^27^ For example, Lufthansa Group and Swiss International Air Lines have applied shark-skin-inspired AeroSHARK films to Boeing 777 fuselages and nacelles, reporting fuel savings of approximately 1% per flight.^28^

It is hypothesized that the disparity between expected performance and real-world observed performance stems from the fact that experimental drag reductions are obtained under narrowly controlled and optimized flow conditions, while flow regimes in real-world applications are typically variable.^29^ As such, this constraint limits their practicality in systems with variable flow, such as drinking water distribution systems, where fluctuations in consumer demand result in continuously changing flow regimes.^30,31^ Therefore, to understand the origins of these limitations and guide the development of more robust and effective surface designs, it is necessary to first examine the physical mechanisms underlying riblet-induced drag reduction.

The mechanism by which riblets reduce drag has been extensively studied through experiments,^32–35^ and simulations.^36–41^ In turbulent flows, skin friction primarily results from coherent near-wall structures known as streamwise vortices.^42–44^ These vortices align with the main flow direction and typically form counter-rotating pairs.^42,45^ As they travel downstream, they also move laterally across the flow, interacting with the wall and adjacent vortices.^46^ These interactions produce high-energy sweep and ejection events that are responsible for much of the momentum transfer to the wall and the resulting drag.^47^

It has been reported in numerical studies that when riblet geometries are carefully tuned to the flow conditions, they can prevent streamwise vortices from penetrating the grooves by confining them above the riblet tips.^36,48^ This confinement creates a low-shear region within the grooves, concentrating high shear near the tips and thereby reducing the overall surface area exposed to intense turbulence.^49–51^ The effectiveness of this mechanism depends on geometric parameters such as riblet spacing, height, and thickness.^20,52,53^ Among these parameters, spacing plays a particularly critical role, as it must be scaled to the size of the dominant near-wall vortices, which in turn depends on the local flow velocity.^36,49^ When the riblet spacing is correctly scaled with the characteristic vortex diameter, the vortices are prevented from entering the grooves and remain above the riblet tips. This configuration minimizes turbulent momentum transfer into the riblet valleys and results in lower frictional resistance. When the spacing becomes too large or the flow velocity increases, the size of vortices become smaller relative to the spacing between riblets and therefore riblets enter the grooves.^54^ This increases surface interaction and leads to higher drag. Riblets must therefore be scaled to a narrow band of vortex sizes to prevent penetration.

High-fidelity direct numerical simulations (DNS) have visually confirmed the lifting of near-wall vortices across different Reynolds numbers. Ananth et al. investigated the behavior of riblets on low-pressure turbine blades and their effectiveness in reducing profile loss under various streamwise loading conditions.^55^ Their study showed that riblets promote earlier transition compared to smooth surfaces under all tested loadings. Moreover, riblets were found to reduce turbulence intensity by displacing vortices away from the wall, particularly at low Reynolds numbers. Investigations into various riblet geometries have shown that sharp-tipped, V-shaped profiles are more effective than curved ones in deflecting near-wall vortical structures.^56^ This enhanced deflection contributes to greater viscous drag reduction, particularly under varying pressure gradient conditions. Further analysis demonstrated that riblet height also plays a critical role, with excessive height increasing flow blockage. These findings underscore the importance of tailoring riblet dimensions to local flow conditions and highlight the sensitivity of optimal riblet performance to flow variations, such as those encountered in transitional regimes.

Streamwise vortices in turbulent boundary layers span a wide range of scales, reflecting the multiscale nature of turbulence and the coexistence of vortices of different size.^26,57^ Goldstein and Tuan,^58^ demonstrated that large turbulent structures interacting with riblets can generate smaller-scale vortices. The presence of these induced small-scale vortices near the wall enhances cross-flow fluctuations and vertical mixing, which in turn contributes to increased drag. Conventional riblets, designed with a single characteristic spacing, can only lift vortices of a specific size, leaving smaller-scale structures largely unaffected.^51^ These smaller vortices can penetrate the grooves, increasing surface interaction and ultimately leading to higher drag. Notably, attempts to reduce riblet spacing to block smaller vortices introduce new challenges. A smaller spacing increases the number of riblets per unit area, which in turn enlarges the total surface area exposed to high-shear flow.^59^ Thus, both oversizing and under sizing the riblet spacing can degrade performance. These design constraints underscore the need for alternative design strategies that can maintain high levels of drag reduction across variable and realistic flow conditions.

In response to the limitations of conventional riblet designs, researchers have explored new riblet arrangements and configurations to enhance their interaction with turbulent flow structures. Design variations including trapezoidal or scalloped cross sections,^60^ spatially varying spacing,^61,62^ and sinusoidal riblet arrangements,^63–65^ have shown measurable performance improvements. However, such reported improvements often apply only under specific conditions and do not translate into general performance gains.

Although geometry modifications such as tapering, asymmetric profiles, and gradient heights can help reduce the surface area exposed to high shear, they are not sufficient on their own to suppress vortices of varying sizes. Because turbulent flows contain vortices across a range of scales,^58^ an effective drag-reducing surface must incorporate riblet elements that interact with multiple vortex sizes. This requires a hierarchical arrangement, in which smaller riblets are embedded between larger ones to manage the multiscale character of turbulence.

To address this, recent studies have explored hierarchical riblet designs that incorporate secondary riblets between primary ones to suppress both large and small vortices.^35,66,67^ For instance, Ou et al.,^67^ proposed a hierarchical nested riblet surface featuring embedded secondary riblets. Their simulations showed that the secondary riblets reduce energy transfer between the turbulent flow and the hierarchical surface, thereby enhancing drag reduction performance. While this represents an advancement in multiscale riblet design, the reported design was based on uniform riblet height and thickness across each level. This choice increases the surface area exposed to high shear, which may limit performance gains under practical conditions. A recent critical review of patterned surface technologies for water applications have emphasized the importance of linking surface geometry to flow behavior and highlighted knowledge gaps in multiscale surface−flow interactions.^25^

The limitations of existing riblet designs in achieving enhanced drag reduction across a wide range of flow conditions highlight the need for surfaces that can interact with turbulence at multiple scales while minimizing the area exposed to high shear. This study proposes a new design strategy to meet both objectives simultaneously. By targeting the multiscale character of streamwise vortices and incorporating geometry that reduces the shear-exposed area, our proposed designs aim to achieve more consistent and higher drag reduction across a wide range of flow conditions. Importantly, while most prior studies have focused on external flow on a plate or channel flow, this work evaluates performance in pipe flow, a geometry representative of industrial systems such as drinking water distribution networks. In such geometries, pipe curvature introduces additional complexities, and our earlier findings indicated that drag-reduction performance is sensitive to both pipe diameter and riblet dimensions.^68^ The proposed riblet designs were fabricated using 3D printing and tested for their hydrodynamic performance in pipe flow under varying flow condition. Drag reduction was evaluated across a range of Reynolds numbers (*Re*), and the influence of nondimensional riblet spacing (*s* ^+^) was analyzed. Furthermore, a conceptual model based on vortex−riblet interactions was developed to explain the mechanisms underlying the performance differences among the designs.

## 2 Methods

### 2.1 Design Evolution: From Uniform to Hierarchical Step-Shaped Riblets

The evolution of the proposed riblet designs was guided by the need to overcome the performance and scaling limitations observed in the literature. The starting point is the Conventional Riblet (CR), which features uniform, flow-aligned primary riblets inside the pipe as conceptually shown in Figure 1a. While effective under controlled conditions, this design is optimized to interact primarily with larger-scale vortices and does not suppress smaller vortices, due to its relatively wide spacing. As a result, its performance is limited to a narrow range of flow conditions where the vortex size matches the riblet spacing, making it highly sensitive to variations in turbulence structure and Reynolds number.^29^

**Figure 1:**
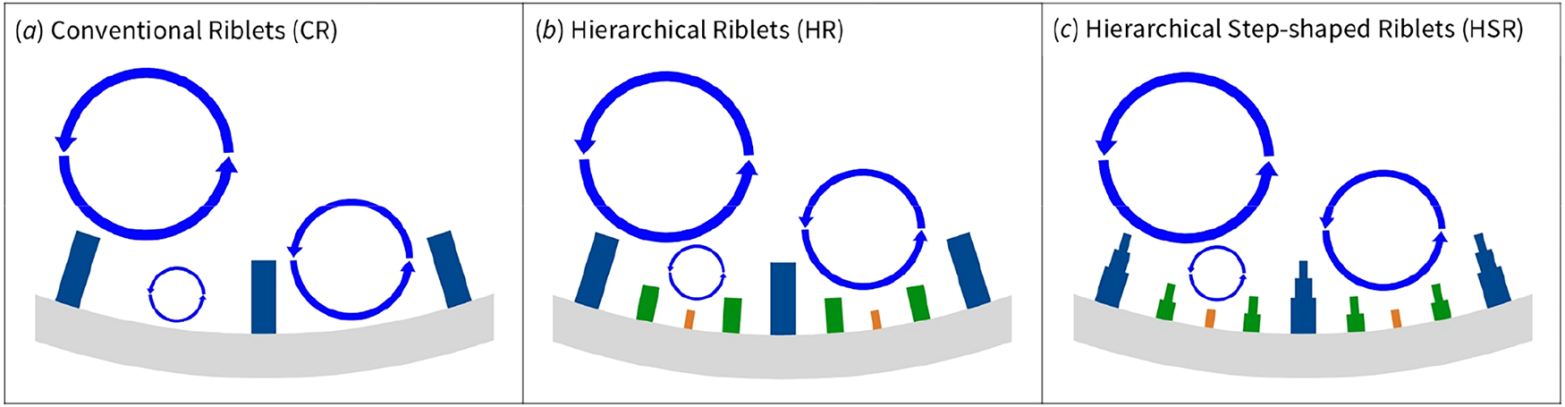
Schematic representation of the riblet design evolution. (a) Conventional Riblet (CR) with uniform primary riblets aligned to the flow direction. (b) Hierarchical Riblet (HR) featuring secondary and tertiary riblets nested between the primary blades to target vortices of different scales. (c) Hierarchical Step-shaped Riblet (HSR) with progressively tapered riblet tips to minimize shear-exposed surface area while maintaining multiscale vortex interaction.

To broaden the effective range of vortex interaction, the Hierarchical Riblet (HR) design introduces secondary and tertiary riblets nested between the primary blades (Figure 1b). This multiscale arrangement is intended to engage vortices of different sizes, enhancing adaptability to the multiscale nature of turbulence and varying flow regimes. However, the increased geometric complexity also enlarges the surface area exposed to high shear, which can partially offset the drag reduction benefit.^69^

In an attempt to overcome this functional weakness, the concept is further developed into the Hierarchical Step-shaped Riblet (HSR) by progressively reducing riblet thickness at each level Figure 1c. This configuration retains the multiscale riblet arrangement of the HR design but introduces a progressive tapering in riblet thickness at each level. By reducing the surface area at the riblet tips, the HSR design minimizes high-shear exposure while maintaining effective multiscale vortex interaction. This feature directly addresses the concerns raised in earlier studies, such as the work of Goldstein et al.,^49^ which emphasized the need to manage small-scale turbulent structures without enlarging the surface area.

The evolution of the riblet design from CR to HR and ultimately to HSR reflects a targeted response to the physical mechanisms and limitations identified in the literature. The HSR design embodies an integrated strategy that combines geometric hierarchy with surface area efficiency, aiming to deliver robust drag reduction in variable pipe flows.

### 2.2 Sample Design

The three riblet configurations described in Section 2.1, namely the CR, the HR, and the HSR, were implemented on the inner surface of a pipe as shown in Figure 2. Their geometric details, including spacing, height, and thickness, are presented in Figure 2a–c, respectively.

**Figure 2:**
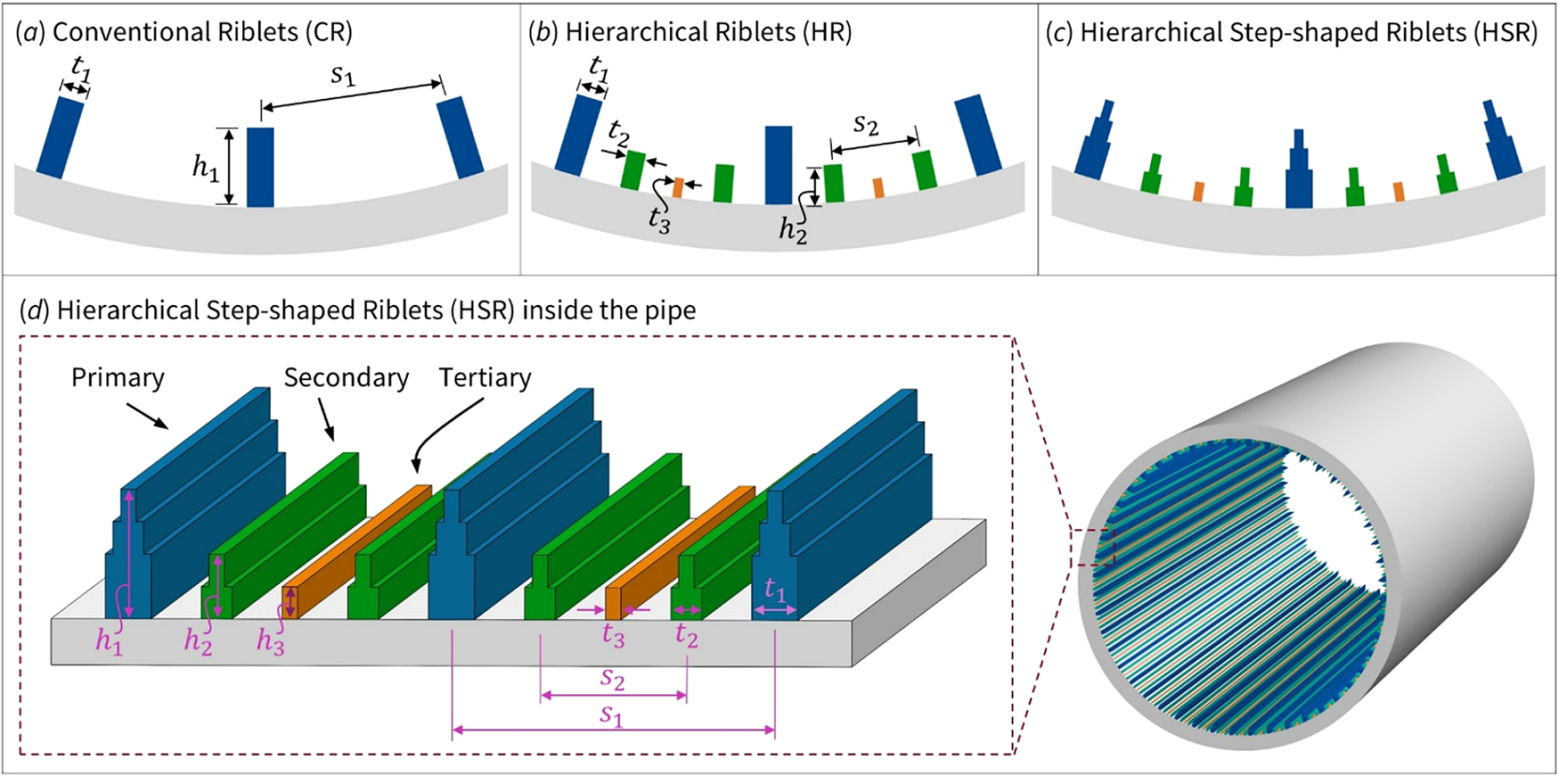
Schematic of the design of the riblet profile. Design evolution and geometry of riblet surfaces. (a) Conventional Riblet (CR) design, consisting only of primary riblets. (b) Hierarchical Riblet (HR) design, which adds secondary and tertiary riblets between primary riblets to interact with vortices of varying sizes. (c) Hierarchical Step-shaped Riblet (HSR) with progressively reduced riblet thickness to minimize shear-exposed area. (d) Surface profile and dimensional parameters of the HSR design implemented within the pipe. The dimensions include riblet spacing (*s*), height (*h*), and thickness (*t*), with subscripts 1, 2, and 3 corresponding to primary, secondary, and tertiary riblets, respectively. Riblet thickness is reduced in multiple steps for primary and secondary riblets, while it remains constant for tertiary riblets.

Figure 2d presents the surface profile and key dimensions of the HSR design implemented within the pipe with effective inner diameter of 20 mm and surface roughness of 2 μm. The parameters *s*, *h*, and *t* represent the spacing, height, and thickness of the riblets, respectively. Subscript numbers indicate the riblet level, where 1 refers to the primary, 2 to the secondary, and 3 to the tertiary riblets. The spacing between primary riblets (*s*_1_) and secondary riblets (*s*_2_) was set to 1.2 and 0.5 mm, respectively. The tertiary riblet is positioned midway between the primary riblets, at a distance equal to half of *s*_1_ to maintain geometric symmetry. The height of the primary riblets (*h*_1_) was set to 0.4*s*_1_, and the secondary riblets to *h*_2_ = 0.4*s*_2_. The height of the tertiary riblet (*h*_3_) was defined as 0.5*h*_2_. The riblet thickness values were set to *t*_1_ = 0.2*s*_1_, *t*_2_ = 0.1*s*_1_, and *t*_3_ = 0.05*s*_1_. The selected geometric ratios were chosen based on established riblet scaling studies, which indicate that optimal drag reduction is typically achieved when the riblet height-to-spacing ratio falls within *h*/*s* ≈ 0.4−0.5.^70,71^ These ratios not only align with prior findings but were also selected to ensure feasible fabrication and structural integrity using the available 3D printing method.

In the primary riblet of the HSR design, the thickness was reduced in two steps: from *t*_1_ at the base, to *t*_2_ at the second level, and *t*_3_ at the top. In the secondary riblet, the thickness was reduced in a single step from *t*_2_ at the base to *t*_3_ at the tip. The thickness of the tertiary riblet remained constant at *t*_3_ across all designs. The riblet thickness was constrained by manufacturing limitations, as producing thin, tall riblets directly from the base was not feasible. Such geometries were prone to deformation or breakage, so extreme tapering ratios were avoided. A thicker base and moderate tapering were adopted to ensure structural stability and reliable fabrication. The final dimensions were selected to balance mechanical integrity with flow control effectiveness, guided by established riblet scaling principles..^72,73^ The 3D printer used had a resolution of approximately 25 μm in *x*−*y* plane, which is of the order of the smallest riblet features. To ensure geometric fidelity, all printed samples were inspected using optical microscopy. While the printed riblets remained stable during flow testing, their long-term mechanical durability under cleaning or operational conditions was not evaluated in this study.

### 2.3 Sample Fabrication and Characterization

The designs were fabricated using a masked stereolithography (MSLA) 3D printer (Prusa SL1S) with Prusament Resin Tough Rich Black, a UV-sensitive, epoxy-based resin selected for its durability and fabrication quality. Due to printer height limitations, each pipe segment was designed to be approximately 135 mm long. Multiple segments were joined to achieve the total pipe length required for fully developed flow conditions (see Supporting Information, Section S1).

The quality and integrity of the fabricated riblet structures were evaluated using optical microscopy. Figure 3 displays representative microscope images of the three riblet designs fabricated via masked stereolithography (MSLA). The imaging was performed using a 4K digital microscope (Keyence VHX-7000 series, Japan). Figure 3a shows the CR design with uniformly spaced primary riblets. Figure 3b illustrates HR configuration, where secondary and tertiary riblets are added between the primary riblets to target turbulent structures of different scales. Figure 3c presents the HSR design, which features a progressive reduction in riblet thickness. The images confirm the precision and resolution achieved through MSLA 3D printing, as well as the structural fidelity of each riblet configuration.

**Figure 3:**
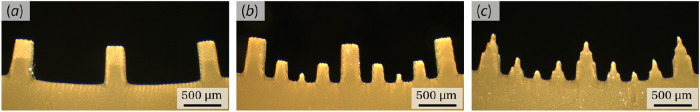
Cross-sectional optical microscopy images of 3D-printed riblets using masked stereolithography. (a) Conventional Riblet (CR) design, consisting of uniformly spaced primary riblets, serving as the baseline configuration. (b) Hierarchical Riblet (HR) design, in which secondary and tertiary riblets are added between the primary riblets to engage near-wall vortices of different sizes and improve flow control. (c) Hierarchical Step-shaped Riblet (HSR) design, with riblet thickness gradually reduced to minimize shear-exposed area and enhance drag reduction performance.

### 2.4 Processing of Experimental Data

To enable comparison with previous studies and evaluate the influence of geometric parameters, all key variables were nondimensionalized using wall units. Following the convention introduced by Bechert et al.,^20^ the nondimensional riblet spacing (*s* ^+^) was defined as

1 $$s^{+}=\frac{su^{*}}{\vartheta }$$

where *s* is the dimensional riblet spacing, ϑ is the kinematic viscosity of water, and u* is the friction (shear) velocity. The friction velocity was determined as

2 $$u^{*}={\left(\frac{\tau_{w}}{\rho }\right)}^{1/2}$$

with τ_w_ representing the wall shear stress and ρ the fluid density. The wall shear stress was calculated from the measured pressure drop across the pipe using the following relation^74^

3 $$\tau_{w}=\frac{D}{4}\frac{\Delta p}{\Delta L}$$

Here, Δ*p* is the pressure drop over the length Δ*L*, and *D* is the equivalent effective diameter of the pipe. The diameter was defined as that of an ideal, smooth circular pipe with the same cross-sectional area, determined from the volume of water required to fill the test section.

A similar nondimensionalization was applied to riblet height (*h* ^+^) and thickness (*t* ^+^), and vortex diameter (*d* ^+^) using the same viscous scaling. Superscript “+” denotes normalization with respect to the viscous length scale (ϑ/u*).

The Darcy friction factor (*f*) was calculated from pressure drop data using the following relation^75^

4 $$f=\frac{\Delta p}{\Delta L}\frac{{D^{5}\pi }^{2}}{8{\rho Q}^{2}}$$

where *Q* is the volumetric flow rate. The drag change percentage was then calculated to compare the performance of riblet-lined pipes (*f*_rib_) with the reference pipe (*f*_ref_)­

5 $$\mathrm{dragchange}\;\%=\frac{f_{\mathrm{rib}}-f_{\mathrm{ref}}}{f_{\mathrm{ref}}}\times 100$$

Negative values indicate drag reduction, while positive values indicate increased resistance.

To account for variations in flow regime and velocity, the Reynolds number was defined as

6 Re=V̅Dϑ=4QπDϑ

where *V̅* is the bulk flow velocity, *Q* is the volumetric flow rate, and ϑ is the kinematic viscosity of water.

To capture the full drag transition regime, from reduction to increase, the flow rate was systematically varied in each pipe, allowing control over both Re and *s* ^*+*^.

### 2.5 Flow Control and Pressure Drop Measurement

To evaluate the hydrodynamic performance of the test pipes, a custom-built water flow loop was established for controlled experimentation. The system consisted of a constant-headwater tank, an inlet flow conditioning section, a horizontal test section, and an outlet reservoir. Flow regulation was achieved via a globe valve installed upstream of the test section, allowing precise adjustment of flow rate and control over a wide range of Reynolds numbers. Pressure drop measurements were acquired across a 0.82-m-long test section using a high-precision differential pressure transmitter (Yokogawa EJA110E). The flow loop was designed to promote fully developed flow conditions prior to the measurement region, minimizing entrance effects and ensuring repeatable data. Additional details on the flow system design and calibration procedures are provided in Supporting Information (Sections S2 and S3).

### 2.6 Validation of Experimental Data with Theoretical Predictions

To evaluate the reliability of the experimental setup and validate the experimental methodology and data acquisition process, the friction factor (*f*) data for the 3D-printed untextured pipe was compared with theoretical predictions based on the Colebrook correlation^76^

7 1fpred=−2.0log(e/D3.7+2.51Refpred)

Where *f*_pred_ is the predicted friction factor, *e* is the surface roughness, *D* is the pipe diameter, and Re is the Reynolds number. Since this equation is implicit in *f*, an iterative method was used to solve for the friction factor.

To assess the accuracy of model predictions relative to experimental observations, the Mean Absolute Error percentage (MAE%) was calculated. This metric represents the average absolute deviation between predicted and measured friction factor values, expressed as a percentage of the predicted values. It is defined as

8 $$\mathrm{MAE}=\frac{1}{n}\sum_{i=1}^{n}\frac{\left|f_{\mathrm{pred},i}-f_{\mathrm{meas},i}\right|}{f_{\mathrm{pred},i}}\times 100$$

Where *f*_pred_ and *f*_exp_ denote the predicted and experimentally measured friction factor values, respectively, and *n* is the number of data points considered.

Figure 4 compares the experimental results with the theoretical predictions. The data shows excellent agreement, with an MAE of approximately 1%. Across the tested Reynolds number range, the percentage error consistently remained within ± 1%, confirming the accuracy of the measurement system. These results validate the robustness of the experimental setup and support the reliability of the data for subsequent analysis.

**Figure 4:**
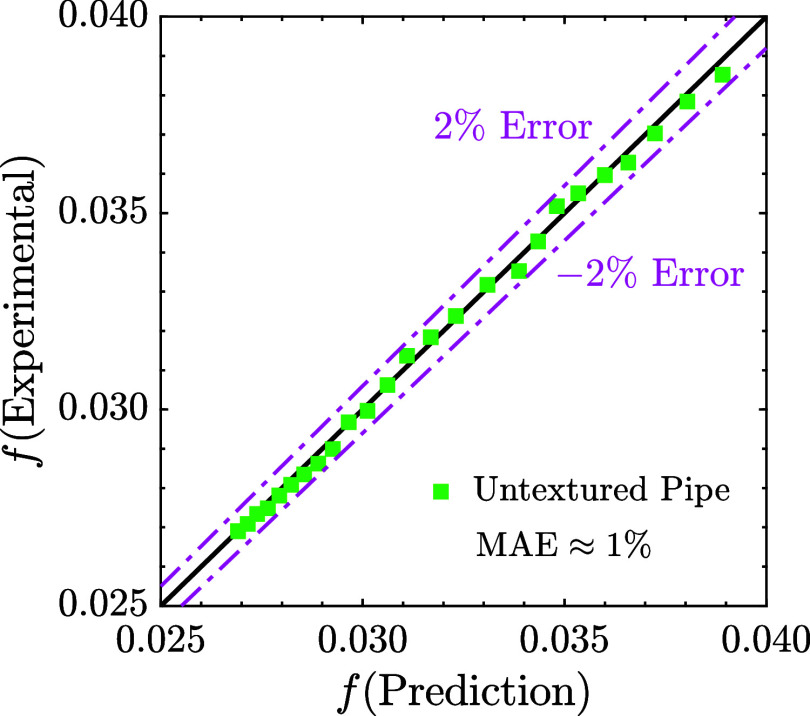
Comparison between experimentally measured friction factor values for the untextured reference pipe and theoretical predictions based on the Colebrook correlation.^76^ Green square markers represent the data for the reference untextured pipe. Dotted dashed purple lines indicate ± 2% error margins. The results show close agreement, with a Mean Absolute Error (MAE%) of approximately 1%, confirming the accuracy of the experimental measurements.

## 3 Results and Discussion

### 3.1 Drag Change Analysis Based on Nondimensional Riblet Spacing (*s* ^+^)

To investigate the effect of riblet geometry on hydrodynamic drag, a series of experiments were conducted using three riblet configurations: CR, HR, and HSR. Figure 5 presents the drag change percentage as a function of nondimensional riblet spacing (*s* ^+^), plotted with respect to two parameters: primary riblet spacing (*s*_1_^+^) and secondary riblet spacing (*s*_2_^+^). This representation facilitates a more detailed evaluation of how each hierarchical feature contributes to overall drag reduction performance.

**Figure 5:**
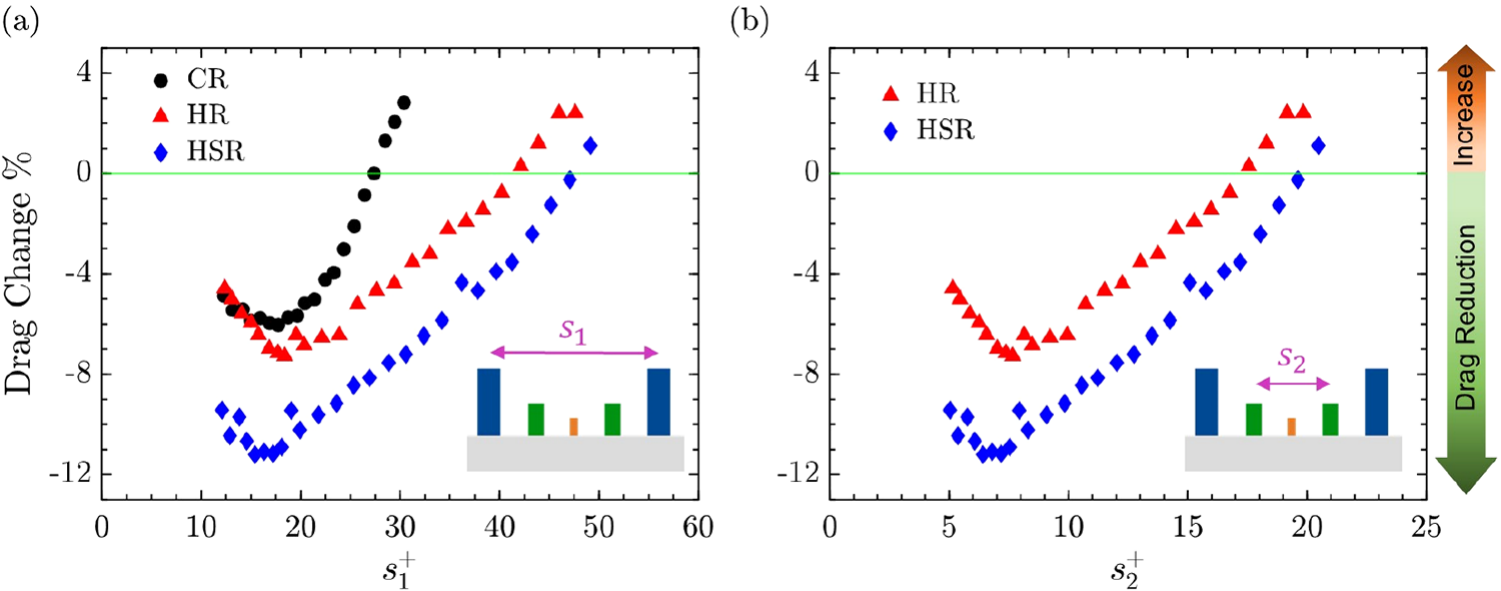
Hydrodynamic performance of three riblet design as a function of *s* ^+^. Drag change percentage was plotted as a function of (a) primary riblet spacing *s*_1_^+^ for CR (black circles), HR (red triangles), and HSR (blue diamonds) and (b) secondary riblet spacing *s*_2_^+^ for HR and HSR designs. Negative values represent drag reduction, while positive values indicate drag increase. HSR consistently shows more drag reduction across a wider range of spacings. Measurement uncertainty across all data points is within ±1%, as discussed in Section 2.6.

As seen in Figure 5a, all three riblet configurations followed a distinct V-shaped trend with increasing *s*_1_^+^. Initially, drag reduction improves as spacing increases, reaching a minimum value before gradually declining. This behavior occurs when optimal spacing keeps vortices above the riblet tips and reduces momentum transfer, leading to lower drag.^49^ As the spacing increases, vortices begin to penetrate the grooves, leading to higher drag.^51^ For the CR design, the maximum drag reduction of approximately 6% compared to a smooth pipe occurred near *s*_1_^+^ ≈ 18. Beyond this point, as *s* ^+^ continued to increase, the drag-reducing capability of the CR design diminished, and the drag change eventually became positive, indicating that the textured pipe surface exhibited greater flow resistance than the untextured pipe. This trend aligns with previously documented behavior in conventional riblet studies.^20,51,52^ As discussed in Section 2.1, the CR design is optimized to interact with larger-scale near-wall vortices. However, this large-scale spacing does not suppress smaller-scale turbulent structures, which penetrate the grooves and contribute to increased momentum transfer and drag.^58^

The HR design exhibited a similar trend but achieved a greater peak drag reduction of approximately 7.3% at a comparable riblet spacing. Notably, the decline in performance beyond this minimum point was more gradual than that of the CR design. Consequently, HR maintained negative drag change values over a substantially wider range of *s*_1_^+^. This outcome confirms the design objectives outlined earlier in Section 2.1, where HR was proposed to engage vortices of varying scales. The sustained drag reduction across a wider range of spacing values compared to CR indicates that the multiscale riblet configuration maintains effective turbulence control under varying flow conditions and is less sensitive to spacing variations. This behavior is also in line with previously reported numerical simulations which indicate that hierarchical structures can be expected to accommodate the multiscale nature of turbulent vortices.^67^

The HSR configuration showed the highest drag reduction among all designs, reaching nearly 11.2% at *s*_1_^+^ ≈ 16. In addition, it maintained negative drag change values across the broadest range of spacings, indicating consistent performance across different spacing conditions. Notably, the upper threshold for effective drag reduction was observed at *s*_1_^+^ ≈ 47 for the HSR design. Beyond this point, none of the tested configurations outperformed the smooth pipe. The results reinforce the conceptual foundation presented in Section 2.1. The HSR retained multiscale vortex interaction while further reducing shear-exposed surface area through stepwise thinning of the riblet tips. The combination of improved drag reduction and reduced sensitivity to spacing demonstrates that combining multiscale riblet geometry with stepwise thinning results in a more effective and robust design than either feature alone.

To further investigate the role of hierarchical structuring, Figure 5b presents the drag change percentage as a function of secondary riblet spacing *s*_2_^+^ for the HR and HSR configuration. While the analysis in Figure 5a focused on the primary riblet spacing *s*_1_^+^, this alternative perspective isolates the influence of secondary riblet elements by plotting the results against their respective nondimensional spacing *s*_2_^+^. In both cases, the physical geometry remains fixed and changes in *s* ^+^ arise solely from variations in flow rate. This approach allows evaluation of how different riblet levels contribute to drag behavior under varying flow conditions. As seen, both HR and HSR exhibited well-defined minima in drag change, with HR achieving peak drag reduction near *s*_2_^+^ ≈ 7.5 and HSR reaching its lowest value near *s*_2_^+^ ≈ 6.5.

The drag reducing range of HR was observed within approximately 5< *s*_2_^+^ < 17, and for HSR within 5 < *s*_2_^+^ < 20. Notably, these *s*_2_^+^ ranges are smaller than the typical nondimensional diameter of near-wall streamwise vortices, which is generally reported as *d* ^+^ ≈ 20−30.^36,51,77^ When the spacing between riblets is smaller than the vortex diameter, vortices tend to remain elevated above the riblet tips rather than penetrating the grooves. This reduces near-wall momentum transfer and contributes to drag reduction.^36,51^

Notably, the spacing range 12 < *s*_2_^+^ < 20 aligns with the extended drag-reducing regime observed for *s*_1_^+^ between 30 and 47. This correspondence suggests that although the HR and HSR designs are effective at relatively large *s*_1_^+^, their extended drag reduction is primarily driven by the secondary riblets. The secondary spacing remains within a favorable range for suppressing smaller-scale vortices and limiting groove penetration. This observation validates the core principle behind hierarchical riblet configurations where riblet elements of varying size are used to manage the coexistence of turbulent structures across scales.^67^

Examining the relationship between *s*_1_^+^ and *s*_2_^+^ highlights the importance of multiscale geometric tuning in hierarchical riblet design. Incorporating *s*_2_^+^ as a performance metric alongside *s*_1_^+^ enhances design strategies and provides deeper insight into how secondary riblets interact with turbulent structures across different scales.

### 3.2 Overall Hydraulic Behavior

The practical suitability of the proposed riblet configurations under operational flow conditions, such as those in water distribution networks, was evaluated based on Reynolds number, as shown in Figure 6. Unlike the nondimensional spacing analysis in Section 3.1, which focuses on local flow−structure interactions, this analysis captures the overall hydraulic behavior across the Reynolds number spectrum.

**Figure 6:**
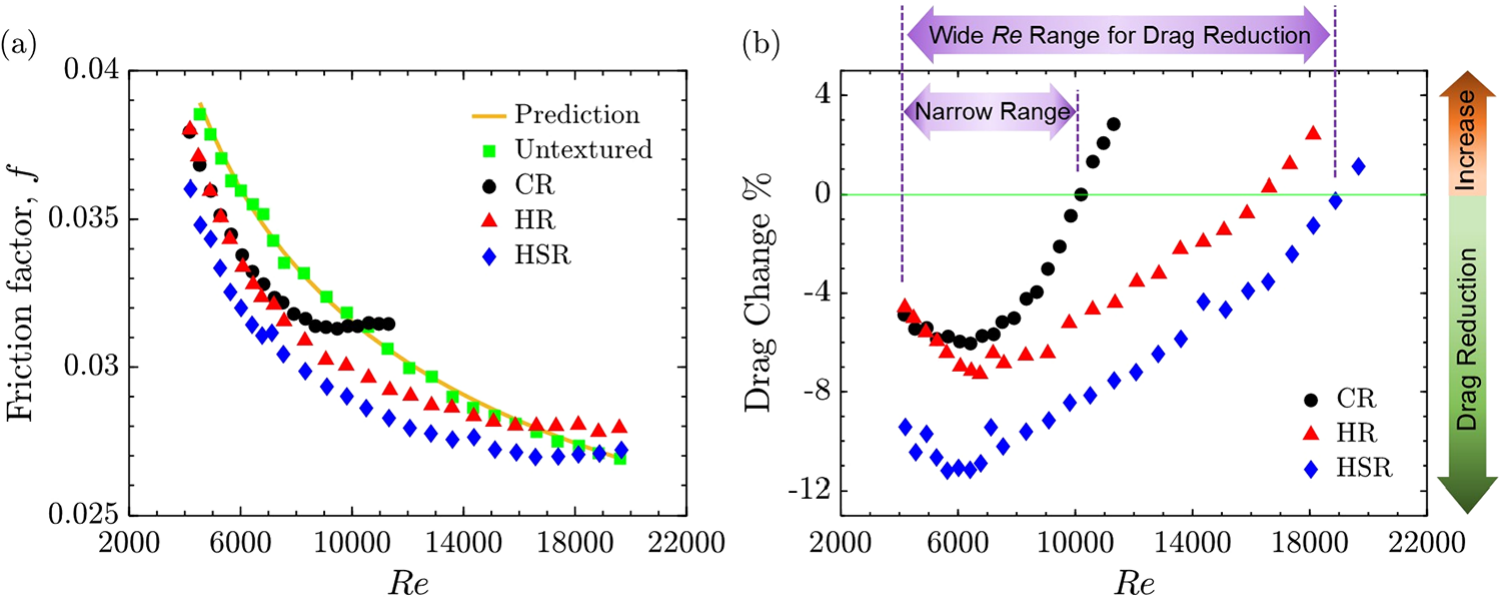
Reynolds number effect on overall hydraulic behavior. (a) Friction factor (*f*) as a function of Reynolds number (Re). Experimental results are shown for the Conventional Riblet (CR) design (black circles), Hierarchical Riblet (HR) design (red triangles), and Hierarchical Step-shaped Riblet (HSR) design (blue diamonds), alongside the untextured reference pipe (green squares). Theoretical predictions based on the Colebrook correlation,^76^ are represented by the orange line. (b) Hydrodynamic performance of three riblet design as a function of Re. HSR exhibits the largest drag reduction and sustains improved performance across the broadest *Re* range.

Following an approach similar to the classical Moody diagram,^78^ Figure 6a shows that, compared to the untextured pipe, the CR design reduced the friction factor over a Reynolds number range of approximately 4200 to 10,000. With further increases in *Re*, the friction factor began to rise and eventually exceeded that of the untextured pipe. The HR design followed a similar trend to CR at lower Re, particularly up to around 7200. With further increase in Re, HR maintained lower friction values than CR over an extended range, up to approximately 16,000. Beyond this point, its friction factor increased and eventually surpassed that of the untextured pipe. The HSR design consistently exhibited the lowest friction factor values among all configurations. It sustained reduced friction across the widest Reynolds number range, from approximately Re ≈ 4200 to approximately 19,000. As Re increased, the HSR curve gradually converged with that of the untextured pipe. Beyond Re ≈ 19,000, its friction factor slightly surpassed that of the untextured pipe. These results suggest that around the Reynolds number of 10,000, the performance of CR begins to deteriorate relative to the smooth pipe. Beyond this threshold, hierarchical riblet designs become increasingly important for maintaining drag reduction at higher flow rates. It is important to note that the Reynolds number ranges reported here correspond specifically to the pipe diameter used in this study and may vary with different pipe geometries or flow configurations. As demonstrated in our previous study,^70^ riblet performance is highly dependent on both pipe diameter and riblet geometry.

These results suggest that the HR and HSR designs are more tolerant to fluctuations in Reynolds number and flow rate, maintaining drag reduction across a wider Reynolds number range than the CR design, as shown in Figure 6b. This makes them well suited for applications with variable operating conditions, such as drinking water distribution systems or industrial cooling networks.^31^

### 3.3 Discussion on the Effect of Vortex-Riblet Interaction on Drag Reduction

To explain the observed drag performance trends across different riblet designs, a conceptual model was developed based on vortex−riblet interactions and is illustrated in Figure 7. This model highlights how streamwise vortices of varying diameters interact with conventional and hierarchical riblet surfaces under different Reynolds number regimes.

**Figure 7:**
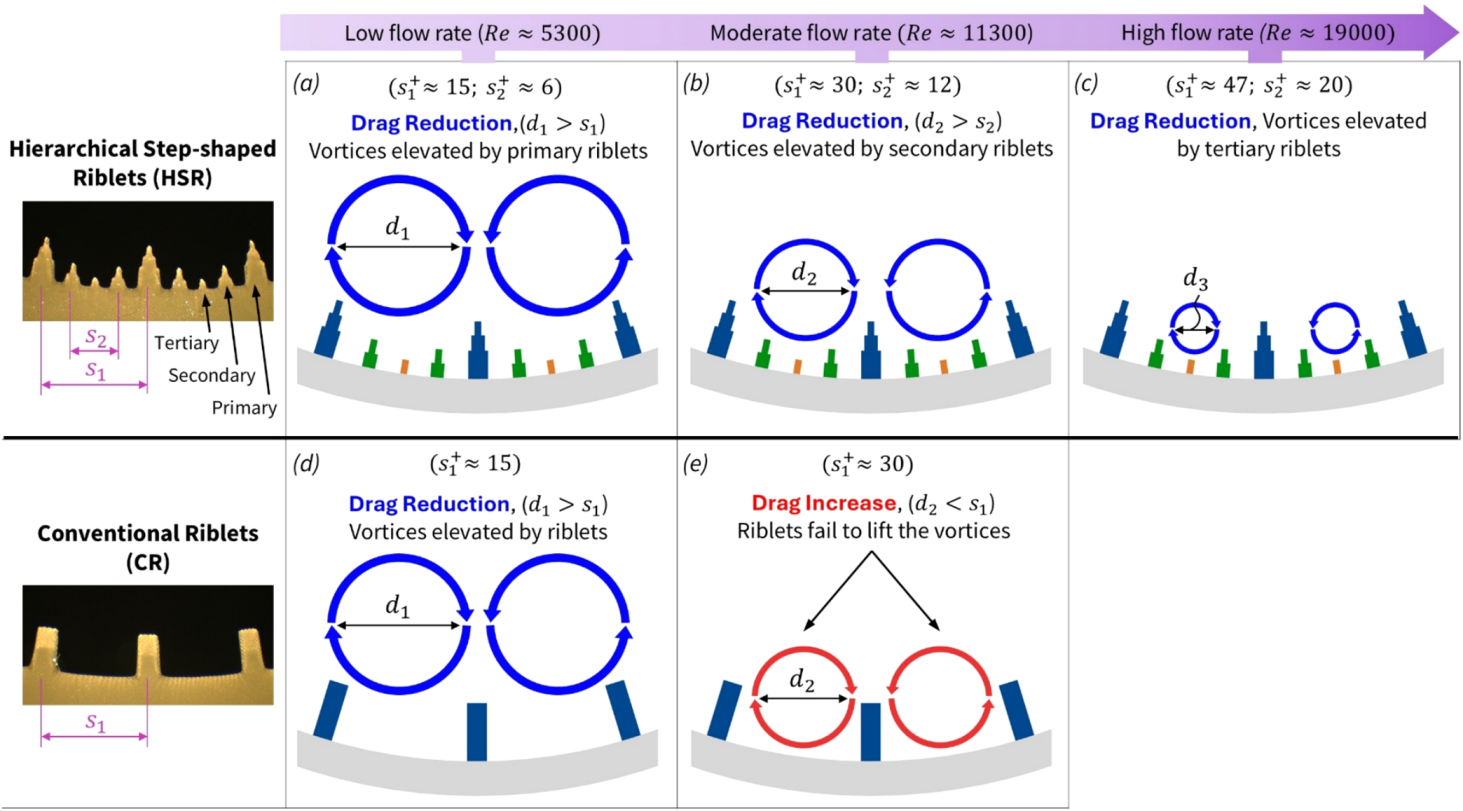
Conceptual model illustrating the interaction between streamwise vortices and riblet geometries under varying Reynolds number (Re). (Top row) The Hierarchical Step-shaped Riblet (HSR) design maintains effective vortex lifting across low, moderate, and high Re by successively engaging primary, secondary, and tertiary riblets with vortices of decreasing diameter (*d*_1_ > *d*_2_ > *d*_3_). (Bottom row) The Conventional Riblet (CR) design exhibits effective vortex lifting only at low *Re* when vortex diameter *d*_1_ is bigger than riblet spacing *s*_1_ (d^+^ > *s* ^+^). At higher Re, as vortex size decreases, the single-scale riblets fail to lift the vortex, leading to increased surface interaction and drag.

In the HSR design (top row), drag reduction is sustained across a wide range of Reynolds numbers due to its ability to interact with vortices of varying scales through a hierarchical riblet structure. This performance is closely linked to the well-established observation that streamwise vortex diameter decreases as Reynolds number increases.^42^ This relationship can be understood through the nondimensional vortex diameter, *d* ^+^ = (du*)/ϑ which typically ranges between 20 and 30 in wall-bounded turbulent flows.^36,51,77^ For a constant pipe diameter and fluid viscosity (water), increasing flow rate raises the friction velocity u*, thereby reducing the dimensional vortex diameter *d*. This scale reduction explains why different riblet levels within the HSR design become effective at different Reynolds number, as visually demonstrated by high-fidelity DNS simulations showing the lifting of near-wall vortices across different Reynolds number.^55,56^ The following analysis illustrates how this multiscale interaction enables the HSR surface to maintain drag reduction under varying flow conditions.

At Re ≈ 5300, as shown in Figure 7a, the primary riblets with *s*_1_^+^ ≈ 15 are well tuned to lift large vortices (*d*_1_), effectively minimizing vortex−wall interaction and preventing penetration of vortices into the grooves. As the flow rate increases to Re ≈ 11,300 (Figure 7b), the vortex diameter decreases while *s*_1_^+^ increases to 30. Since *s*_1_^+^ becomes comparable to or slightly larger than the average *d* ^+^ ≈ 20−30, the effectiveness of the primary riblets in lifting vortices is reduced. However, the presence of secondary riblets with *s*_2_^+^ ≈ 12 at this regime, interacts with the smaller vortices (*d*_2_) and lifts the vortices at this scale, resulting in an extended drag reduction regime. At even higher Reynolds numbers (Figure 7c), the tertiary riblets engage the smallest vortices (*d*_3_), enabling continued suppression of near-wall turbulence. This staggered vortex-lifting mechanism allows the HSR surface to accommodate changing vortex scales and maintain drag reduction across a wide range of flow conditions.

In contrast, the CR design (bottom row) suppresses near wall turbulence across a much narrower flow range. At *Re* ≈ 5300 (Figure 7d), it performs comparably to HSR, as the primary spacing *s*_1_^+^ ≈ 15 remains below *d* ^+^ ≈ 20−30, enabling effective vortex lifting. However, as Re increases and vortex diameter decreases (Figure 7e), *s*_1_^+^ increases to ≈30, exceeding the typical vortex diameter. Without secondary or tertiary riblets, vortices penetrate the grooves, increasing wall interaction and shear stress, ultimately leading to drag increase.

The superior performance of the HSR design can be attributed to its multiscale architecture, with each riblet level engaging vortices of different sizes. This hierarchical coordination lifts a broader spectrum of turbulent structures away from the wall, reducing momentum transfer and sustaining drag reduction under variable flow conditions. In contrast, the single-scale CR surface operates effectively only within a narrow Reynolds number range, limiting its practical applicability. While the riblet levels in HR and HSR are designed to engage vortices of different characteristic sizes, potential interference between adjacent levels cannot be fully excluded. However, the consistent drag reduction observed in hierarchical designs suggests that such possible interactions do not compromise performance. Future studies involving flow visualization or numerical simulations could provide further insight into the interplay between riblet levels and their combined influence on vortex dynamics.

Overall, the multiscale vortex−riblet interaction observed in the HSR configuration provides a robust physical basis for its broader and more stable drag-reducing performance and thus can be applicable in a variety of sectors. It is important to note that, as demonstrated in our previous work,^70^ drag reduction performance in pipe flow depends on both riblet geometry and pipe diameter. Therefore, although the current results offer a strong foundation, applying the same design to other pipe sizes would require re-evaluating the nondimensional riblet parameters to maintain effectiveness.

## 4 Conclusions

This study introduced and experimentally validated a hierarchical riblet design strategy to reduce skin-friction drag in turbulent pipe flows with varying operating conditions. The progression from a conventional riblet configuration (CR) to a multiscale hierarchical design (HR), and ultimately to a step-shaped geometry (HSR), addressed fundamental limitations in flow sensitivity and narrow operational range. The HSR design, featuring tapered multilevel riblets, consistently achieved the greatest drag reduction and maintained performance across the broadest Reynolds number range.

Among the three configurations, the HSR design achieved the highest peak drag reduction of 11.2% and remained effective across a broader Reynolds number range than the other designs. Notably, it sustained favorable drag reduction even at large primary riblet spacings (*s*_1_^+^ ≈ 30−47) by maintaining secondary riblets within an effective vortex-suppressing range (*s*_2_^+^ ≈ 13−20), underscoring the importance of multiscale geometric tuning.

The introduction of *s*_2_^+^ as a complementary design and evaluation parameter alongside *s*_1_^+^ provides a more robust framework for characterizing riblet performance under realistic flow conditions. This enables the design of surfaces that remain effective across a wide range of Reynolds numbers, particularly in variable-flow systems such as drinking water distribution networks. Moreover, the tapered profile implemented in the HSR design demonstrates the feasibility of producing geometrically complex riblets using 3D fabrication techniques.

These results highlight the value of tailoring riblet geometry to the multiscale structure of turbulence while minimizing the surface area exposed to shear. The outcomes provide a basis for designing more effective surface and structural designs and demonstrate the practical potential of hierarchical riblets for energy-efficient fluid transport in systems such as drinking water distribution systems.

## Supplementary Material



## Notes

Pipe design and connection; fluid flow experimental setup; experimental procedure (PDF)
