# Supplementary material for: Hierarchical Riblet Structures for Enhanced Drag Reduction and Broader Operational Range in Water Pipelines
Source: ACS ES T Water. 2025 Sep 5;5(10):6030–40. doi: 10.1021/acsestwater.5c00703 (PMC12519487; doi:10.1021/acsestwater.5c00703)
Supplement: Supplementary file 1 [file ew5c00703_si_001.pdf]

Supplementary Material for

Hierarchical Riblet Structures for Enhanced Drag Reduction and Broader Operational Range in Water Pipelines

Mirvahid Mohammadpour Chehrghani,†,‡ Jamal Seyyed Monfared Zanjani,*,† Doekle Yntema,‡ David Matthews,† Matthijn de Rooij,†

†Faculty of Engineering Technology, University of Twente, 7500AE, Enschede, the Netherland

‡ Wetsus, European Centre of Excellence for Sustainable Water Technology, Oostergoweg 9, 8911 MA Leeuwarden, The Netherlands

* Corresponding author: j.seyyedmonfaredzanjani@utwente.nl

S1. Pipe Design and Connection

To achieve the required pipe length for fully developed flow conditions, the test pipe was fabricated from multiple segments due to the height limitations of the 3D printer. Each segment was designed with interlocking features: a male connector at one end and a female connector at the other. This configuration ensured precise alignment, mechanical stability, and a leak-free seal between segments. **Figure S1a** shows a cross-sectional illustration of the pipe ends, highlighting the integrated connection geometry. **Figure S1b** displays two segments joined using a Tri-Clamp system, which provided secure and repeatable assembly throughout the test setup.

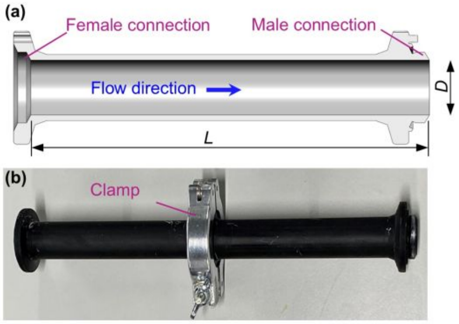

Figure S1. Pipe design and connection system. (a) Cross-sectional view showing the integrated female (inlet) and male (outlet) connectors at each end of the pipe segments, designed to ensure proper alignment and sealing during assembly. (b) Photograph of two connected segments secured using a Tri-Clamp, enabling a stable and leak-tight connection suitable for extended test lengths.

S2. Fluid Flow Experimental Setup

The experimental system used to assess drag reduction performance is shown in **Figure S2**. It comprised four main components: a constant-head water tank, an inlet section, a test section, and a differential pressure measurement system.

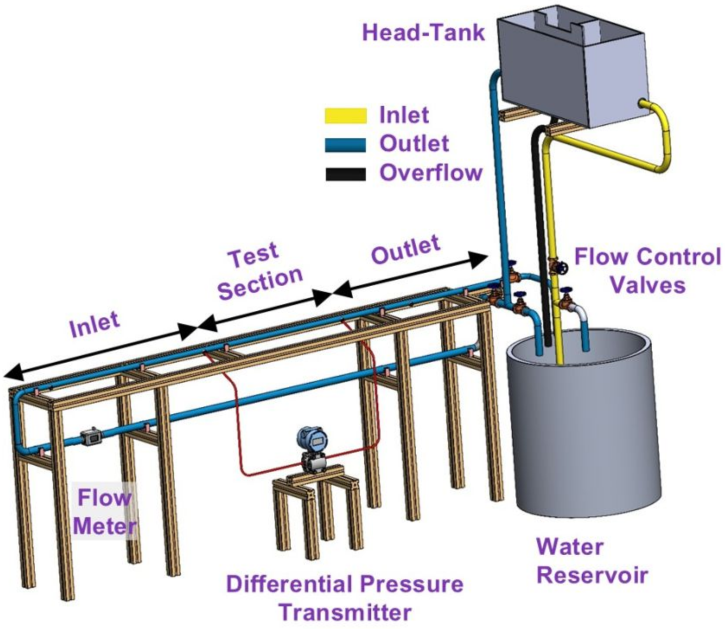

Figure S2. Schematic diagram of the experimental setup used for pressure drop measurements. The diagram illustrates the key components of the open-loop water flow system, including the constant-head tank, inlet and outlet sections, test section, pressure taps, flow regulation valve, flow meter, and differential pressure transmitter. The layout was designed to ensure stable, fully developed turbulent flow and accurate pressure drop readings across the riblet-lined test section.

Accurate pressure drop measurements required careful positioning of the pressure taps and transmitter. Following manufacturer guidelines[1], the pressure taps were mounted at a 30° angle below the horizontal plane. The differential pressure transmitter (Yokogawa EJA110E) was installed below both the pressure taps and the main pipeline. This arrangement ensured a consistent downward slope in the impulse lines, minimizing the risk of air entrapment and improving measurement stability. The pressure drop across a designated segment of the test section was monitored using the EJA110E differential pressure transmitter. The device was configured with a measurement span of 3 kPa, well within its full operating range of 0.5–5 kPa, and offered a high accuracy of 0.055% of span. Water flow rate was continuously recorded using an Endress+Hauser Picomag IO-link electromagnetic flow meter. With a specified measurement error of ±0.8%, the device provided accurate and repeatable flow rate data throughout the experiments.

S3. Experimental Procedure

Tap water from a ground-level reservoir was pumped into a constant-head tank designed to maintain a steady head of 2.6 meters (as shown in **Figure S2**). The tank incorporated an internal separation wall and an overflow mechanism to ensure that the pressure head remained consistent throughout all experiments. This configuration stabilized the flow conditions across tests. To eliminate pump-induced pulsations and ensure a steady flow regime, the system employed gravity-driven flow instead of direct connection to the process piping. Water exiting the tank entered a 4.0-meter-long pipeline equipped with a globe valve for flow regulation, an electromagnetic flow meter, and a thermometer. The flow path included two elbows before reaching the inlet section. The inlet segment measured approximately 1.5 meters in length, corresponding to a length-to-diameter (L/D) ratio of about 57. This extended ratio was selected to ensure hydrodynamically fully developed flow, where the velocity and temperature profiles are stable[2]. This condition is

essential for producing consistent shear stress along the pipe wall and a reliable pressure drop under each flow condition[3]. Although fully developed flow is typically achieved at *L/D* ratios around 10 in standard engineering systems, the larger *L/D* ratio used here provided additional confidence in the flow development. Following the inlet section, water entered the test section, which was approximately 1.6 meters long. Pressure drop measurements were carried out over a designated subsection of about 0.82 meters within this region. After passing through the test section, the flow continued through a 0.9 meters long outlet pipe before returning to the reservoir, completing the open-loop configuration.

Supplementary References

1. *Yokogawa EJX and EJA-E Series Differential Pressure and Pressure Transmitters Installation Manual*.
2. Pritchard, P. J. & Mitchell, J. W. *Fox and McDonald's Introduction To Fluid Mechanics Eighth Edition*. (John Wiley & Sons, 2016).
3. White, F. M. *Fluid Mechanics*. (McGraw-Hill Education, 1979).
